# A hybrid recommendation framework utilizing domain-adaptive RoBERTa embeddings for enhanced personalization in e-commerce

**DOI:** 10.1038/s41598-026-38853-5

**Published:** 2026-03-22

**Authors:** Chour Singh Rajpoot, Varun Tiwari, Santosh Kumar Vishwakarma

**Affiliations:** 1 School of Computer Science and Engineering, Manipal University Jaipur, Rajasthan, 303007 India; 2 Department of Computer Science and Engineering, Gyan Ganga Institute of Technology and Sciences, Jabalpur, Madhya Pradesh 482003 India

**Keywords:** Hybrid recommendation, Collaborative filtering, Attention fusion, Behavioural statistics, Learning-to-Rank, Engineering, Mathematics and computing

## Abstract

With the rapid growth of e-commerce and online platforms, delivering personalised and accurate recommendations remains a challenge due to sparse interaction data and diverse user interests. This paper proposes **HyReC**, a **unique hybrid recommendation framework** that integrates content-based and collaborative filtering while maintaining computational efficiency. Domain-adaptive **RoBERTa embeddings** are used to extract semantic representations from textual content, capturing user and item preferences from descriptions and reviews. A **Deep Neural Network (DNN)** model uses user-item interactions to generate latent behavioural embeddings, which are enriched behavioural **statistical features** such as mean rating, rating variance (standard deviation), interaction frequency, and skewness. Heterogeneous embeddings are fused using a **Bahdanau attention mechanism**, enabling the model to dynamically weight content, collaborative, and statistical signals. The fused representation is then used to generate recommendations through a **Learning-to-Rank layer**, depending on application scale. A model is trained using the **Adam optimiser** to ensure fast convergence and stable performance. Experimental evaluation on the Amazon Baby dataset demonstrates that HyReC achieves superior performance **of 0.15**, **MAE of 0.10**, **MSE of 0.023**, **R² of 0.98**, **Pearson Correlation of 0.99**, **MAPE of 1.5%**, and **F1-score of 0.98**, outperforming state-of-the-art models such as LSTM, RBM + KNN, GNN, and GAT. Experiments on benchmark datasets demonstrate that the proposed framework **improves recommendation accuracy**,** diversity**,** and robustness** compared to baseline models, effectively addressing **data sparsity**,** user interest drift**,** and heterogeneous content**.

## Introduction

Recommendation systems are smart technologies that interpret users’ historical behaviour and preferences to generate tailored recommendations across domains such as e-commerce, education, and entertainment^1^. Their main purpose is to help users wade through copious amounts of information without experiencing information overload and decision fatigue, or confusion, and to enhance streamlined decision-making processes^2^. Many approaches to recommendations, the content-based filtering technique recommends items by matching user profiles to item features, while the collaborative filtering technique uses user–item interaction patterns to find similarities among users and among items^3,4^. Knowledge-based methods, where knowledge graphs are incorporated, achieve a deeper context and semantic relevance, achieve better accuracy, and produce more frequently adaptive and meaningful personalised recommendations^5^.

Hybrid recommendation systems, which combine content-based and collaborative filtering approaches, experience challenges related to high computational complexity, dependence on the quality of features, difficulty in accommodating users’ dynamic preferences, and sensitivity to sparse or incomplete data^6^. Hybrid systems also experience issues with data sparsity, cold-start problems, high computational cost, adaptability to changes in a user’s personal interests, and do so while requiring a large quality dataset to achieve optimum performance^7^. Furthermore, hybrid systems also face difficulties integrating contextual factors, limited scalability, and lower accuracy for systems with insufficient user or item data^8^. Additionally, inaccuracies, overspecialization, scalability, sparsity, and cold-start problems lead to increased computational complexity and reduced adaptability for new users or items, therefore affecting the accuracy and overall performance and efficiencies of the whole system^9^.

Recent developments in recommendation systems have increasingly popularised the combination of content-based knowledge and collaborative filtering for higher accuracy, personalisation, and contextual level of comprehension. The suggested approach combines knowledge graph representation learning and neural collaborative filtering methods, using pretrained embeddings to extract semantics and improve recommendations via matrix factorisation^10^. Graph Neural Networks and word2vec embeddings are utilised to help estimate high-order item relations, creating richness for both collaborative and content filtering to improve accuracy^11^. Deep neural networks are leveraged to increase user personalisation. Another step allows us to use user vocabulary preference features to help overcome cold start and sparsity problems^12^. To improve contextual relevance and accuracy of recommendations, we combine Word2Vec and Bi-LSTM models to analyse textual reviews and interactions between users^13^.

Recommendation systems are an essential component of improving user experience, as they leverage significant amounts of data by filtering and displaying recommendations suitable to the user^14^. Content-based and collaborative filtering methods have been successful, but they often fall short when dealing with issues of data sparsity, cold-start situations, and limited adaptability to user preferences^15^. The paper proposed HyReC, a hybrid recommendation architecture that merges the understanding of semantic user behaviour in a single framework. Using an appropriately trained domain-adaptive RoBERTa embedding combined with deep neural networks and an attention-based mechanism, the models proposed in this framework aim to improve recommendation accuracy and account for contextualised user intents while also improving diversity. This hybrid structure serves to build a more robust and flexible model and is applied in the practice of varying types of e-commerce and online recommendation problems.

The main contribution of this paper is as follows,

- Hybrid Recommendation Domain-Adaptive Embeddings merges content-based and collaborative filtering methods by utilising RoBERTa embeddings for both users and items, addressing challenges related to sparse data and heterogeneous content.
- Attention-Based Fusion of Heterogeneous Signals utilises Bahdanau attention to merge content, collaborative, and behavioural statistical features, enhancing accuracy, diversity, and robustness.
- Efficient and scalable training is achieved through the use of the Adam optimiser and an optional Learning-to-Rank Layer, ensuring fast convergence, stable performance in recommendations, and scalability for delivering quality outcomes.

The structure for this paper is discussed in the introduction for the recommendation systems. Section 2 review of various recommendation models, including content-based, collaborative Filtering. The proposed HyReC framework is detailed, integrating RoBERTa embeddings, Deep Neural Networks for collaborative filtering, behavioural statistics, and Bahdanau attention for fusion, as detailed in Sect. 3. Results from experimental evaluations and benchmark datasets highlight comparisons of baseline models, addressing accuracy, diversity, and robustness in Sect. 4. The conclusion summarises the contributions to recommendation literature, suggests implications of the proposed method, and considers potential future work in Sect. 5.

## Literature survey

In 2025, Alam and Ahmed^16^ investigated DL techniques designed for recommendation systems that will improve accuracy and personalisation. Restricted Boltzmann Machines (RBM) are a method that has been implemented effectively in collaborative filtering to model complex user–item interactions. While recommendation systems using RBMs have good performance, combining them with k-Nearest Neighbour (kNN) provides improved predictive performance through neighbourhood-based similarity, while also tackling traditional recommendation issues associated with sparsity, scalability, and cold start in datasets such as Movielens-1 M.

In 2025, Sedgh et al.^17^ examined that enhanced learning (also referred to as TEL) emphasises the increasing prevalence of deep learning and sentiment-aware recommender systems for improving personalisation. Neural collaborative filtering has gained popularity in modelling complex learner–content interactions, and sentiment analysis, models like BERT capturing emotional feedback. Finally, adaptive learning mechanisms, for example, based on AdaGrad optimisation, excel in generating a more responsive system than the limitations of static and/or emotionally neutral TEL.

In 2025, Gao^18^ proposed that Systems for recommending library books emphasise the improvement of accuracy using deep learning methods and hybrid models. Typical collaborative filtering and content-based approaches suffer from scalability and accuracy issues. While transformer-based architectures have been shown to be strong at providing better contextual relationships and adaptive extreme learning machines are capable of resolving nonlinear feature fusion. Merging both these methodologies improves recommendation performance to a much superior level than conventional recommendation algorithms for large-scale library datasets.

In 2025, Ma^19^ analysed that the application of ML has been effective in learning behaviour analysis and personalised recommendations, specifically in online education recommendation systems. ML models leveraged learning factors such as learning time, frequency of interaction, and performance data to identify patterns of learner habits and preferences. Such intelligent systems provide a level of engagement and adaptability, yielding greater accuracy and personalisation for an online educational environment over traditional static recommendations.

In 2024, Suvarna and Balakrishna^20^ investigated that Recommender systems focus on using DL and an ensemble approach to improve personalisation and trustworthiness. The single-model paradigm has struggled with accuracy in more complex domains like fashion. Research indicates that improved feature extraction and prediction are attained with deep ensemble classifiers by using multiple models such as MobileNet, DenseNet, Xception, and VGG. Including a similarity measure, such as cosine similarity, can refine recommendations of products and applications relating to e-commerce.

In 2024, Saini and Singh^21^ examined that Recommender systems exemplify the efficacy of deep learning algorithms in modelling complicated user-item relationships. Traditional collaborative filtering algorithms are being supplanted by neural-based algorithms, which often use stacked LSTM and attention-based autoencoders, which improve sequences and feature extraction. Self-supervised learning improves adaptability and overall performance, which improves large-scale datasets such as Amazon product recommendations.

In 2024, Ghadami and Tran^22^ explored highlighting hybrid recommender systems, which combine collaborative filtering (CF) and content-based filtering (CBF) to jointly address the limitations of each approach and enhance accuracy. Conventional CF is user-centric, whereas CBF considers item attributes. Empirical results indicate that hybridisation of CF and CBF, utilising deep neural networks and various similarity metrics, including cosine similarity, aids in rating prediction, personalisation, and inherently improves recommendation performance across multiple application areas.

In 2024, Ziaee et al.^23^ proposed that movie recommender systems emphasise graph-based DL models as a way of addressing the shortcomings of conventional collaborative and content-based filtering. Research demonstrates that Graph Neural Networks (GNNs) and Graph Autoencoders (GAEs) are able to exploit heterogeneous relationships between users and movies, while also capturing side information and contextual dependencies. The hybrid graph-based approach significantly improves recommendation accuracy while benefiting from data sparsity and the cold-start problem.

In 2025, Liu et al.^24^ found that Academic recommender systems use a combination of Graph Neural Networks (GNNs) and pretrained Transformers to provide improved recommendations based on text. Content-based or keyword-matching recommendations often overlook contextual understanding of information. By combining Transformers used for semantic representation with Attention Networks (GAT) used to model relationships, journal recommendation accuracy is enhanced, and scholars will have more success aligning research articles with appropriate journals based on the title, abstract, and keywords.

In 2024, Siet et al.^25^ proposed that Movie recommender systems are focusing on the use of DL-based hybrid models to handle historical challenges like data sparsity, scalability, and cold-start problems. Transformer architectures and multi-head attention mechanisms are effective in performing sequential modelling of user behaviour. By implementing user demographic information, clustering methods like K-Means, and multilayer perceptrons, these systems achieve improved personalisation, prediction performance, and diversity in recommendations, which offers a more dynamic and contextually aware movie recommender system.

Huang et al. (2025) propose ColdLLM for cold-start item recommendation using user interactions simulated by a large language model (LLM). The method introduces a related funnel to efficiently narrow down potential users, enhancing scalability. It demonstrates improvements over baseline metrics for Recall and NDCG in cold-item categories, verified through online A/B testing.

Shen et al. (2022), developed by pupilRec is a vision-based recommendation system utilizing variations in pupil size to discern user preferences. It captures implicit emotional feedback beyond traditional mouse clicks and employs time series and neural methods to facilitate recommendations. User studies confirm the system’s accuracy.

Zeng et al. (2022) implemented a GRU model for personalised recommendations, while applying attention mechanisms helps prevent overfitting and emphasises the most relevant user attributes. Experimental results show improved accuracy of the model on benchmark data collections.

Table 1 demonstrates the aim, method, and findings from the existing works.

**Table 1:** Review of the existing works.

Author (Year)	Aim	Method	Advantages	Disadvantages
Alam & Ahmed, 2025^16^	To improve accuracy and personalisation in recommender systems.	Deep Learning-based Collaborative Filtering using Restricted Boltzmann Machines (RBM) combined.	Enhances predictive accuracy; tackles data sparsity, scalability, and cold-start problems.	Increased computational complexity; performance depends on dataset size and quality.
Sedgh et al. 2025^17^	To enhance personalisation in Technology Enhanced Learning (TEL) systems.	Neural Collaborative Filtering (NCF) integrated BERT-based Sentiment Analysis and AdaGrad optimisation.	Captures emotional feedback; improves adaptability and personalisation.	Higher model complexity requires large labelled data for sentiment analysis.
Gao 2025^18^	To improve accuracy in library book recommendation systems.	Transformer architectureAdaptive Extreme Learning Machine (ELM).	Provides contextual understanding; resolves nonlinear feature fusion and scalability issues.	High computational demand; needs large training data.
Ma 2025^19^	To analyse learning behaviour and provide personalised recommendations in online education.	Machine Learning algorithms analyse user activity data (time, frequency, interactions).	Improves adaptability and engagement; accurate learning pattern detection.	May struggle with sparse or incomplete behavioural data.
Suvarna & Balakrishna 2024^20^	To enhance personalisation and reliability in fashion product recommendations.	Deep Ensemble Classifier combining MobileNet, DenseNet, Xception, and VGG, with cosine similarity.	Increases accuracy and robustness; refines recommendations through model diversity.	Computationally intensive; risk of overfitting multiple models.
Saini & Singh, 2024^21^	To improve recommendation performance using deep learning.	Stacked LSTMAttention-based Autoencoder under Self-Supervised Learning.	Better sequential modelling enhances adaptability and feature extraction.	High training cost; requires fine-tuning for large datasets.
Ghadami & Tran, 2024^22^	To improve accuracy through hybrid recommendation systems.	Hybrid model combining Collaborative Filtering (CF) and Content-Based Filtering (CBF) using deep neural networks and cosine similarity.	Enhances personalisation and rating prediction; combines user and item features.	Complexity in model integration; data dependency between CF and CBF.
Ziaee et al. 2024^23^	To improve movie recommendations using graph-based deep learning.	Graph Neural Networks (GNN) and Graph Autoencoders (GAE).	Handles data sparsity; captures heterogeneous and contextual information.	High computation time; requires detailed relational data.
Liu et al. 2025^24^	To improve journal recommendation accuracy in academic research.	Transformer combined Graph Attention Network (GAT) using textual inputs.	Enhances semantic and relational understanding; improves context-aware recommendations.	Complex training may require high-quality text preprocessing.
Siet et al. 2024^25^	To create a dynamic movie recommendation model addressing scalability and cold-start issues.	Transformer architecturemulti-head attention, K-Means clustering, and MLP.	Improves personalisation, prediction, and diversity in recommendations.	High resource requirements; sensitive to clustering parameters.
Huang et al. 2025^26^	Cold-start item recommendation	LLM-based user simulation (ColdLLM)	Strong cold-item performance	High complexity
Shen et al. 2022^27^	Better user preference detection	Pupil-size-based CV model	Captures real emotions	Privacy & scalability issues
Zeng et al. 2022^28^	Improve personalization accuracy	GRU with attention	Reduces overfitting	Poor cold-start handling

### Research gap

Recent innovations in recommendation systems have introduced techniques like deep learning and graph models. However, many advanced approaches remain limited, focusing primarily on collaborative user-item interactions without effectively integrating various signal types. Current models improve contextual representation but lack a unified method for capturing the semantic meanings of products and interactions. Pre-trained language models face challenges due to a lack of domain adaptation, diminishing their generalizability. Additionally, hybrid systems often have complex architectures that hinder interpretability. This work proposes a hybrid recommendation framework that is domain adaptive, combining semantic and collaborative signals with behavioural predictive features to enhance prediction performance and interpretability.

## Proposed methodology

A Hybrid Deep Learning Architecture for Ranking or Classification tasks, utilising the pre-trained Transformer model RoBERTa to enhance text understanding. The input text sequences are tokenised, including special tokens like [CLS] and [SEP]. The RoBERTa Layer serves as a robust feature extractor, generating high-dimensional, contextually rich embeddings for each token, capturing their semantic relationships. These embeddings are then processed by a Classification Head, typically comprised of fully connected Deep Neural Network layers, resulting in a final prediction such as a ranking score or class probability distribution, benefiting from RoBERTa’s language comprehension combined with efficient task-specific predictions from the DNN head on the extracted features. The HyReC framework focuses on Top-K item recommendation as a learning-to-rank task, aiming to develop a scoring function that prioritises relevant items for a user over non-relevant ones. While explicit ratings are used for model training alongside behaviour-based representations, the final recommendations are on ranking relevance rather than direct rating predictions. Although binary relevance and rating prediction are considered for analysing model stability and generalisation, they are not the primary tasks of the framework.

**Fig. 1:**
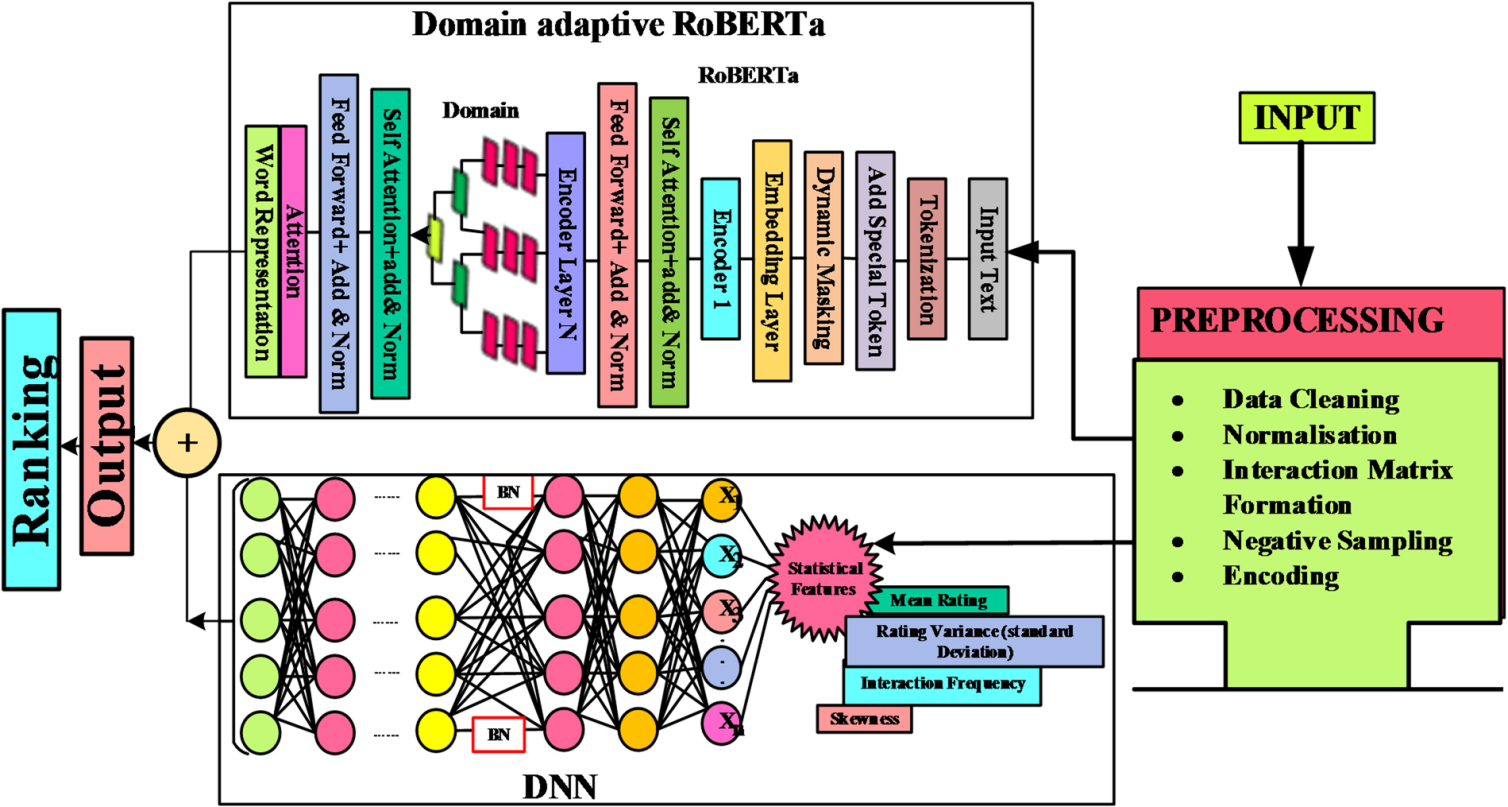
Block diagram for the proposed methodology.

Figure 1 illustrates the layout of a hybrid recommendation system, showing the interactions between Semantic, Collaborative, and Behavioural Modules, along with separate sections for Data Preprocessing and Individual Module Evaluation Actions. The model aims for enhanced top-K ranking accuracy, effective cold-start handling, and interpretable signal importance in recommendations. Its main functions include extracting semantic embeddings, learning collaborative representations from user-item interactions, computing user/item reliability statistics, fusing signals via Learning-to-Rank, and providing an efficient DNN candidate retrieval and re-ranking pipeline. The continuity of the model’s steps ensures that each component builds upon the previous one, leading to improved performance in ranking objectives. Notation for users and items includes: Users denoted as u∈U and items as i∈J. Each item i contains fields such as $\:{\mathrm{T}}_{\mathrm{i}}$(title), $\:{\mathrm{D}}_{\mathrm{i}}$(description), $\:{\mathrm{R}}_{\mathrm{i}}^{(1..\mathrm{m})}$(reviews). Interaction is labelled as $\:{\mathrm{y}}_{\mathrm{u},\mathrm{i}}$ representing either an explicit rating or an implicit signal like a click/purchase. Important dimensions specified include RoBERTa final pooled embedding dimension (d_c), DNN latent dimension. $\:{\mathrm{d}}_{\mathrm{c}}$. DNN latent dim: $\:{\mathrm{d}}_{\mathrm{b}}$. Statistical vector dim: $\:{\mathrm{d}}_{\mathrm{s}}$ And ranking score:$\:{\mathrm{s}}_{\mathrm{u},\mathrm{i}}$.

### Data collection

In the realm of data sharing, each dataset carries a unique narrative, inviting contributors to relay the story behind their data. This narrative should encompass not just the raw numbers and entries within the dataset but also the methodology behind its acquisition, including aspects such as the source and time span covered by the data. Furthermore, it is important to acknowledge the individuals or organisations that contributed to the data collection process, providing thanks and citing relevant past research that has informed one’s work. This communal aspect of data sharing encourages collaboration and reflection within the world’s largest data science community, paving the way for potential inquiries and analyses that are derived from the dataset. Contributors are thus encouraged to consider what questions they wish the community to address using their data. Dataset link is https://www.kaggle.com/datasets/roopalik/amazon-baby-dataset/data.

### Data preprocessing

The preprocessing stage guarantees that the inputs for HyReC’s content and collaborative modules are consistent and devoid of noise, grammar adjustments and sentence verification, enhancing clarity.

- **Data Cleaning and Integration**:

All records $\:(u,i,{y}_{u,i},{t}_{u,i})$are cleaned by removing duplicates and nulls:

1 $$\:{D}^{{\prime\:}}=\left\{\left(u,i,{y}_{u,i},{t}_{u,i}\right)\in\:D{\hspace{0.25em}\hspace{0.05em}}\mid{\hspace{0.25em}\hspace{0.05em}}{y}_{u,i}\ne\:NULL\right\}$$

- **Text Normalisation**: The item text fields, which include title, description, and reviews, are processed by converting them to lowercase, removing HTML, and tokenising them using RoBERTa.

2 $$\:{X}_{i}^{tok}={\mathrm{Tokenizer}}_{RoBERTa}\left({T}_{i}\oplus\:{D}_{i}\oplus\:\mathrm{Top-}k\left({R}_{i}\right)\right)$$

- **Interaction Matrix Formation**: The user-item interactions are defined by.

3 $$\:{R}_{u,i}=\{\begin{array}{cc}{y}_{u,i},&\:\mathrm{if\:interaction\:exists}\\\:0,&\:\mathrm{otherwise}\end{array}$$

A time-based split of 70:10:20 is employed for realistic evaluation.

- **Negative Sampling**: For each positive pair $\:(u,{i}^{+})$, $\:k$negatives are sampled as:

4 $$\:P\left({i}^{-}\right)=\frac{{p}_{i}^{\alpha\:}}{\sum\:_{j}{p}_{j}^{\alpha\:}},\alpha\:\in\:\left[\mathrm{0,1}\right]$$

- **Encoding**: Continuous features are standardised.

5 $$\:\stackrel{\sim}{x}=\frac{x-{\mu\:}_{x}}{{\sigma\:}_{x}}$$

and categorical IDs are numerically encoded. This preprocessing pipeline integrates textual, behavioural, and statistical data to enhance learning stability and generalisation within the HyReC recommendation framework. This preprocessing output is fed to Roberta and a statistical DNN.

### Content representation - domain-adaptive RoBERTa

A model involving several layers: an Embedding Layer transforms tokens into dense word vectors, while Encoder Layers, comprised of Self-Attention, Add & Norm, and Feedforward Network, learn token relationships and apply transformations. Domain-Adaptive Fine-Tuning is implemented for the RoBERTa model to align its understanding of domain-specific language through Masked Language Modelling. A Pooling Layer aggregates token representations into a single vector, and an optional Projection Layer reduces dimensionality for generating content embeddings. These embeddings maintain semantic meaning and sentiment, aiding in item-based filtering by identifying similar items through textual content. The objective is to generate strong semantic vectors for textual metadata and reviews by fine-tuning RoBERTa on a combined domain corpus using Masked Language Modelling (MLM). Optionally, an auxiliary regression/classification head may be added to predict review ratings from review text, aligning semantics ratings. For embedding extraction, the overall embedding for each block of text (title/description/review) will be computed.

6 $$\:{\mathbf{e}}_{\mathrm{X}}={\mathrm{R}\mathrm{o}\mathrm{B}\mathrm{E}\mathrm{R}\mathrm{T}\mathrm{a}}_{\mathrm{p}\mathrm{o}\mathrm{o}\mathrm{l}}\left(\mathrm{X}\right)\in\:{\mathbb{R}}^{{\mathrm{d}}_{\mathrm{c}}}$$

To obtain the item content vector $\:{\mathbf{c}}_{\mathrm{i}}$, aggregate

7 $$\:{\mathbf{c}}_{\mathrm{i}}=\mathrm{P}\mathrm{r}\mathrm{o}\mathrm{j}\left(\frac{1}{\mid\:{\mathrm{S}}_{\mathrm{i}}\mid\:}\sum\:_{\mathrm{X}\in\:{\mathrm{S}}_{\mathrm{i}}}{\mathbf{e}}_{\mathrm{X}}\right),\mathrm{P}\mathrm{r}\mathrm{o}\mathrm{j}:{\mathbb{R}}^{{\mathrm{d}}_{\mathrm{c}}}\to\:{\mathbb{R}}^{{\mathrm{d}}_{\mathrm{c}}^{{\prime\:}}}$$

Where $\:{\mathrm{S}}_{\mathrm{i}}$= chosen set (title + description + top-k reviews). Proj is a small feed-forward layer (optional), $\:{\mathrm{d}}_{\mathrm{c}}^{{\prime\:}}$often set to 256.

**Fig. 2:**
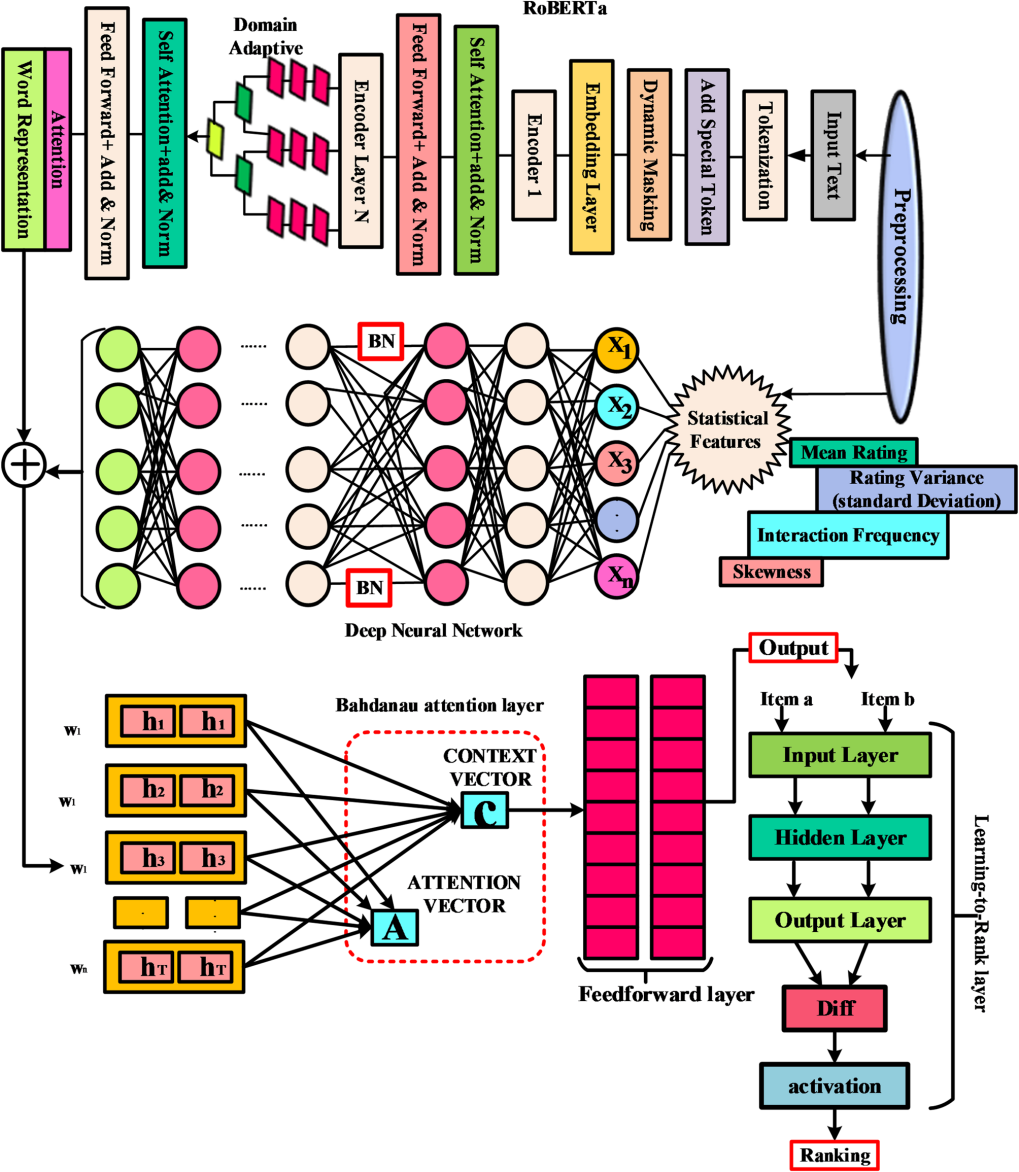
Architecture for the proposed methodology.

Figure 2 shows a multi- architecture for ranking items using both textual and statistical data. It includes Text Processing with a RoBERTa-based model for semantic representation extraction, Feature Integration via a DNN for analyzing statistical features, an Attention Mechanism utilizing Bahdanau attention to generate context vectors, and Ranking Logic involving a Feedforward layer and a “Learning-to-Rank” component that assesses items in pairs to establish their order, culminating in a numerical ranking through a Diff calculation and activation function.

### Collaborative representation -DNN latent embeddings

The collaborative module, based on Deep Neural Networks (DNN), analyses interaction data to identify user-item co-preferences and behavioural patterns. It consists of the following layers: the Input Layer, which combines user embedding vectors, item embedding vectors, and statistical features; Hidden Layers that apply ReLU activation, dropout, and batch normalisation to learn interaction patterns. The Output Layer generates predicted user-item interaction scores.

The purpose is to capture collaborative co-preference patterns.

- Embedding layers: $\:{\mathbf{u}}_{\mathrm{u}}\in\:{\mathbb{R}}^{{\mathrm{d}}_{\mathrm{u}}}$for user $\:\mathrm{u}$, $\:{\mathbf{v}}_{\mathrm{i}}\in\:{\mathbb{R}}^{{\mathrm{d}}_{\mathrm{i}}}$for item $\:\mathrm{i}$.
- Concatenate $\:{\mathbf{z}}_{\mathrm{u},\mathrm{i}}=[{\mathbf{u}}_{\mathrm{u}};{\mathbf{v}}_{\mathrm{i}};\mathrm{(side-features)}]$and feed to a fully connected network:

8 $$\:{\mathbf{b}}_{\mathrm{u},\mathrm{i}}={{\upvarphi\:}}_{\mathrm{L}}\left({\mathbf{W}}_{\mathrm{L}}{{\upvarphi\:}}_{\mathrm{L}-1}\right(\cdots\:{{\upvarphi\:}}_{1}({\mathbf{W}}_{1}{\mathbf{z}}_{\mathrm{u},\mathrm{i}}+{\mathbf{b}}_{1})\cdots\:{\hspace{0.17em}})+{\mathbf{b}}_{\mathrm{L}}),$$

where $\:{\upvarphi\:}$are nonlinearities (ReLU), dropout, and batch normalisation apply. The network outputs a collaborative vector. $\:{\mathbf{b}}_{\mathrm{u},\mathrm{i}}\in\:{\mathbb{R}}^{{\mathrm{d}}_{\mathrm{b}}}$. Alternatively, compute separate user and item behavioural embeddings. $\:{\mathbf{b}}_{\mathrm{u}},{\mathbf{b}}_{\mathrm{i}}$. For explicit ratings: minimise MSE

9 $$\:{\mathcal{L}}_{\mathrm{M}\mathrm{S}\mathrm{E}}=\frac{1}{\mid\:\mathcal{D}\mid\:}\sum\:_{(\mathrm{u},\mathrm{i})\in\:\mathcal{D}}({\mathrm{r}}_{\mathrm{u},\mathrm{i}}-{\widehat{\mathrm{r}}}_{\mathrm{u},\mathrm{i}}{)}^{2}$$

where $\:{\widehat{\mathrm{r}}}_{\mathrm{u},\mathrm{i}}={\mathrm{f}}_{\mathrm{c}\mathrm{o}\mathrm{l}\mathrm{l}\mathrm{a}\mathrm{b}}({\mathbf{u}}_{\mathrm{u}},{\mathbf{v}}_{\mathrm{i}})$. Implicit feedback employs binary cross-entropy, enabled by DNN to identify collaborative signals separate from semantic text features.

### Behavioural statistical features (interpretable)

The explicit signals minimal variance to summarise user and item behaviours for each user and item. Key statistics to capture user tendencies include:

Mean rating (µ_u_), which indicates the user’s average preference,

10 $$\:{{\upmu\:}}_{\mathrm{u}}=\frac{1}{{\mathrm{n}}_{\mathrm{u}}}{\sum\:}_{\mathrm{j}=1}^{{\mathrm{n}}_{\mathrm{u}}}{\mathrm{r}}_{\mathrm{u},\mathrm{j}}$$

Rating variance (σ_u_²), reflecting the consistency of their ratings,

11 $$\:{{\upsigma\:}}_{\mathrm{u}}^{2}=\frac{1}{{\mathrm{n}}_{\mathrm{u}}}{\sum\:}_{\mathrm{j}}({\mathrm{r}}_{\mathrm{u},\mathrm{j}}-{{\upmu\:}}_{\mathrm{u}}{)}^{2}$$

Interaction frequency (f_u_) measures user engagement intensity $\:{\mathrm{f}}_{\mathrm{u}}={\mathrm{n}}_{\mathrm{u}}$(count).

Skewness (skew_u_), indicating the tendency of ratings towards high or low scores,

12 $$\:{\mathrm{s}\mathrm{k}\mathrm{e}\mathrm{w}}_{\mathrm{u}}=\frac{1}{{\mathrm{n}}_{\mathrm{u}}}{\sum\:}_{\mathrm{j}}{\left(\frac{{\mathrm{r}}_{\mathrm{u},\mathrm{j}}-{{\upmu\:}}_{\mathrm{u}}}{{{\upsigma\:}}_{\mathrm{u}}}\right)}^{3}$$

The statistics also factor in recency and frequency of user interactions to capture time-based effects. The derived information is standardised as $\:{\mathbf{s}}_{\mathrm{u},\mathrm{i}}=\mathrm{S}\mathrm{t}\mathrm{d}\left(\left[{\mathbf{s}}_{\mathrm{u}};{\mathbf{s}}_{\mathrm{i}};\mathrm{interaction\:features}\right]\right)\in\:{\mathbb{R}}^{{\mathrm{d}}_{\mathrm{s}}},\:$which involves normalisation through zero mean unit variance scaling and outlier clipping at certain percentiles. These statistics serve as prior information for modelling user activity levels and assist in cold-start situations or regularisation of data. Overall, this framework aims to quantify user behaviour while managing variations and interactions precisely.

### Attention-based fusion (Bahdanau additive)

An adaptively weighted content, collaborative, and statistical vectors for each (user, item) query.

- Query vector $\:\mathbf{q}={\mathbf{b}}_{\mathrm{u}}$(user behavioural embedding) or concatenation $\:\mathbf{q}=[{\mathbf{b}}_{\mathrm{u}};{\mathbf{u}}_{\mathrm{u}}]$.
- Keys/values: $\:\{{\mathbf{k}}_{1}={\mathbf{c}}_{\mathrm{i}},{\mathbf{k}}_{2}={\mathbf{b}}_{\mathrm{i}},{\mathbf{k}}_{3}={\mathbf{s}}_{\mathrm{u},\mathrm{i}}\}$with values $\:{\mathbf{v}}_{\mathrm{j}}={\mathbf{k}}_{\mathrm{j}}$(or projected).

Bahdanau attention equations, for each key $\:\mathrm{j}$:

13 $$\:{\mathrm{e}}_{\mathrm{j}}={\mathbf{v}}_{\mathrm{a}}^{\mathrm{\:}}\mathrm{t}\mathrm{a}\mathrm{n}\mathrm{h}({\mathbf{W}}_{\mathrm{q}}\mathbf{q}+{\mathbf{W}}_{\mathrm{k}}{\mathbf{k}}_{\mathrm{j}}+{\mathbf{b}}_{\mathrm{a}})$$

14 $$\:{{\upalpha\:}}_{\mathrm{j}}=\frac{\mathrm{e}\mathrm{x}\mathrm{p}\left({\mathrm{e}}_{\mathrm{j}}\right)}{\sum\:_{\mathrm{t}}\mathrm{e}\mathrm{x}\mathrm{p}\left({\mathrm{e}}_{\mathrm{t}}\right)}$$

Fused representation:

15 $$\:{\mathbf{f}}_{\mathrm{u},\mathrm{i}}=\sum\:_{\mathrm{j}}{{\upalpha\:}}_{\mathrm{j}}{\mathbf{v}}_{\mathrm{j}}\in\:{\mathbb{R}}^{{\mathrm{d}}_{\mathrm{f}}}$$

Interpretability in this context is represented by the α_j-values, which supply a most dominant per-instance signal. Additionally, the emphasises that additive attention is both data-efficient and robust when it comes to combining heterogeneous modalities.

Ranking head & loss functions

16 $$\:{\mathrm{s}}_{\mathrm{u},\mathrm{i}}={\mathbf{w}}_{\mathrm{s}}^{\mathrm{\:}}{\upvarphi\:}({\mathbf{W}}_{\mathrm{s}}{\mathbf{f}}_{\mathrm{u},\mathrm{i}}+{\mathbf{b}}_{\mathrm{s}})$$

To generate a ranking score and optimise corresponding metrics, the score calculation involves integrating a feed-forward scoring head feed integration vector. This process enables the effective evaluation and adjustment of ranking outcomes. BPR Loss focuses on maximising the score difference between positive and negative items based on implicit feedback. Hinge Loss establishes a margin between positive and negative scores to enhance pairwise ranking. Listwise Loss applies softmax-based probabilities to optimise ranking metrics, like NDCG, for item lists.

Total training loss (with regularisation):

17 $$\:\mathcal{L}={\mathcal{L}}_{\mathrm{r}\mathrm{a}\mathrm{n}\mathrm{k}}+{{\uplambda\:}}_{{\uptheta\:}}\parallel\:{\uptheta\:}{\parallel\:}_{2}^{2}+{{\uplambda\:}}_{\mathrm{a}\mathrm{t}\mathrm{t}}{\mathcal{L}}_{\mathrm{a}\mathrm{t}\mathrm{t}\_\mathrm{r}\mathrm{e}\mathrm{g}}$$

where $\:{\mathcal{L}}_{\mathrm{r}\mathrm{a}\mathrm{n}\mathrm{k}}$is chosen loss (BPR/listwise), $\:{\uptheta\:}$ are model weights, and attention regularisation encourages sparsity or entropy control of $\:{\upalpha\:}$. Ranking losses enhance training alignment final top-K metrics more effectively than pointwise MSE. The system is optimised for ranking-type loss functions, which are effective for Top-K recommendations. Pointwise loss functions (e.g., RMSE, MAE) for comparison with previous studies and auxiliary analysis.

## Results and discussions

The research focuses on predicting ratings through regression analysis, using RMSE and MAE as primary performance measures, supplemented by MSE, R2, Variance Explained, Pearson Correlation, MaxError, and MAPE for stability and accuracy analysis. A cross-validation method was employed, splitting user interactions into 80% for training and 20% for testing, with experiments repeated across various random seeds. Results indicated that the tested model outperformed the baseline on all metrics, achieving the lowest RMSE (0.15 ± 0.01) and MAE (0.10 ± 0.01), alongside the highest R2 (0.98 ± 0.01) and Pearson Correlation (0.99 ± 0.01). The Top-K analysis corroborated its effectiveness as a recommendation tool, with a Recall and F1 Score of 0.98 ± 0.01, and all performance differences were statistically significant at *p* < 0.05. This dataset includes 17,168 users, 6,782 items, and 56,950 user reviews, each consisting of a rating and comments about the item. The extensive data allows for diverse review analysis to assess the quality of a recommendation system. The model incorporates an embedding dimension of 64, utilises a batch size of 32, and was trained over 100 epochs or until an early stopping criterion, employing the Adam optimiser with a learning rate of 0.0001.

### Dataset description

The Amazon Baby Dataset https://www.kaggle.com/datasets/roopalik/amazon-baby-dataset/data provides a diverse range of product reviews and ratings that are specific to baby- and childcare-related products. The dataset contains user-generated text reviews, numeric ratings, timestamps, and product identifiers for a wide range of baby categories, such as diapers, toys, feeding, and so on. The dataset used by researchers and practitioners to explore problems such as sentiment analysis, recommendation systems, and purchasing behaviour for baby products, specifically. The rich structure of both textual and numeric data, leveraged for tasks such as rating prediction, review classification, and trending, makes this dataset an excellent dataset to understand consumer preference and product performance from the baby care perspective.

### Performance evaluation

The proposed model’s performance evaluation outperforms the existing machine learning and graph-based architectures. It is seen to consistently outperform models, such as LSTM, RBM + KNN, GNN, and GAT, in terms of higher accuracy, precision, and robustness on different evaluation metrics. The proposed framework overcomes challenges such as data imbalance, prediction error, and reduced correlation to ensure that the obtained results are consistent and reliable. It shows balanced performance on different metrics of RMSE, MAE, MSE, R², Explained Variance, Pearson Correlation, MAPE, Recall, and F1-score, which further confirms its efficiency in maintaining prediction stability and classification quality. It stands as a robust and optimised methodology for performance-oriented predictive analytics. Table 2 shows the Comparative performance evaluation of different predictive models.

**Table 2:** Comparative performance evaluation of different predictive models.

Model	LSTM	RBM + kNN	GNN	GAT	Proposed
RMSE	1.22 ± 0.03	1.33 ± 0.04	1.91 ± 0.05	2.56 ± 0.06	0.15 ± 0.01
MAE	0.90 ± 0.02	1.06 ± 0.03	1.53 ± 0.04	2.27 ± 0.05	0.10 ± 0.01
R²	0.45 ± 0.02	0.50 ± 0.02	0.55 ± 0.02	0.60 ± 0.02	0.98 ± 0.01
MSE	1.49 ± 0.08	1.76 ± 0.09	3.63 ± 0.15	6.54 ± 0.20	0.02 ± 0.01
Explained Var	0.45 ± 0.02	0.50 ± 0.02	0.55 ± 0.02	0.60 ± 0.02	0.98 ± 0.01
Max Error	3.50 ± 0.14	4.00 ± 0.16	5.65 ± 0.22	4.75 ± 0.19	0.50 ± 0.05
Pearson Corr	0.65 ± 0.02	0.75 ± 0.02	0.80 ± 0.01	0.82 ± 0.01	0.99 ± 0.01
MAPE	12.0 ± 0.5	10.5 ± 0.4	15.2 ± 0.6	18.0 ± 0.7	1.5 ± 0.2
Recall	0.81 ± 0.02	0.84 ± 0.02	0.86 ± 0.01	0.88 ± 0.01	0.98 ± 0.01
F1	0.82 ± 0.02	0.84 ± 0.02	0.87 ± 0.01	0.88 ± 0.01	0.98 ± 0.01

Table 2 illustrates regression analysis for predicting ratings utilising RMSE and MAE as primary metrics, supplemented by MSE, R2, Variance Explained, Pearson Correlation Coefficient, MaxError, and Mean Absolute Percentage Error to assess model stability and accuracy. A cross-validation method was used, partitioning data into 80% for training and 20% for testing, with multiple experiments for reproducibility. The findings indicate that the model exceeded baseline performance across all metrics, achieving the lowest RMSE (0.15 ± 0.01) and MAE (0.10 ± 0.01), alongside the highest R2 (0.98 ± 0.01) and Pearson Correlation (0.99 ± 0.01). Top-K analysis further validated its effectiveness as a recommendation tool, with a Recall and F1 Score of 0.98 ± 0.01, showing significant performance differences at the *p* < 0.05 level.

**Fig. 3:**
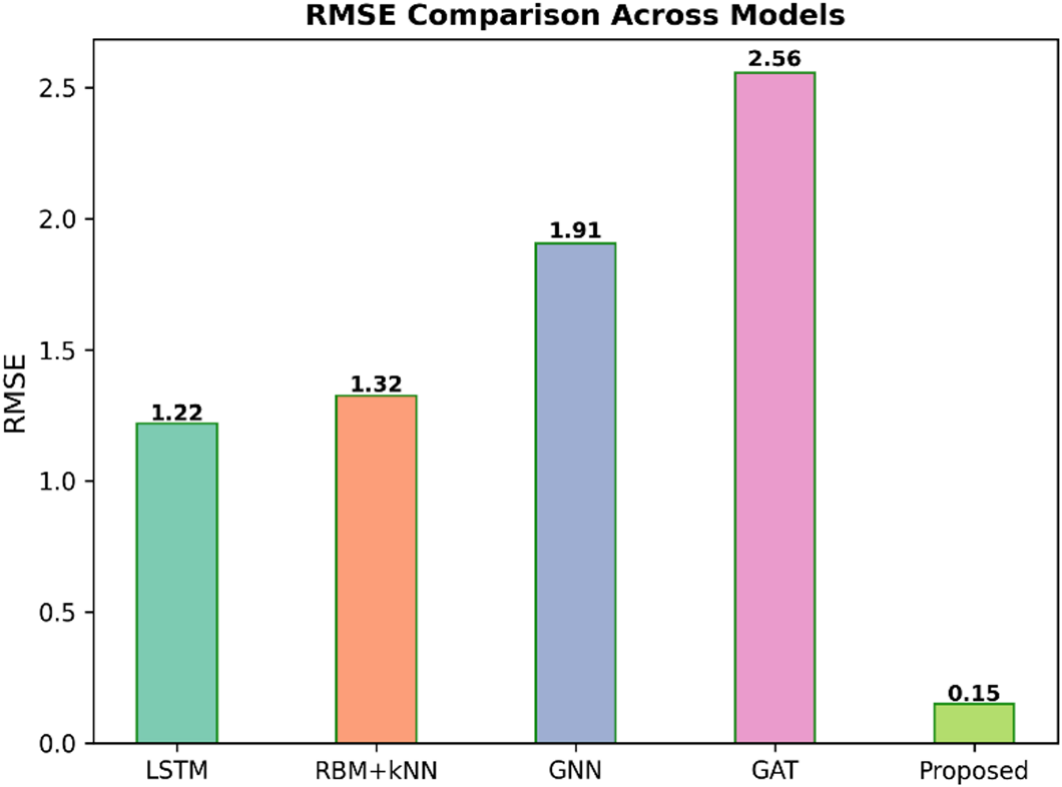
RMSE comparison across models.

Figure 3 shows that the Root Mean Square Error (RMSE) across five different predictive models, such as LSTM, RBM + kNN, GNN, GAT, and the proposed model. The lower RMSE value means the better the model performance. The Proposed model is the best with an RMSE of 0.15, which is much better than the rest. LSTMan RMSE of 1.22, followed by RBM+KNNan RMSE of 1.32, GNNan RMSE of 1.91, and the worst performance of an RMSE of 2.56. These results imply that the proposed model is superior to the existing methods in the accuracy of prediction.

**Fig. 4:**
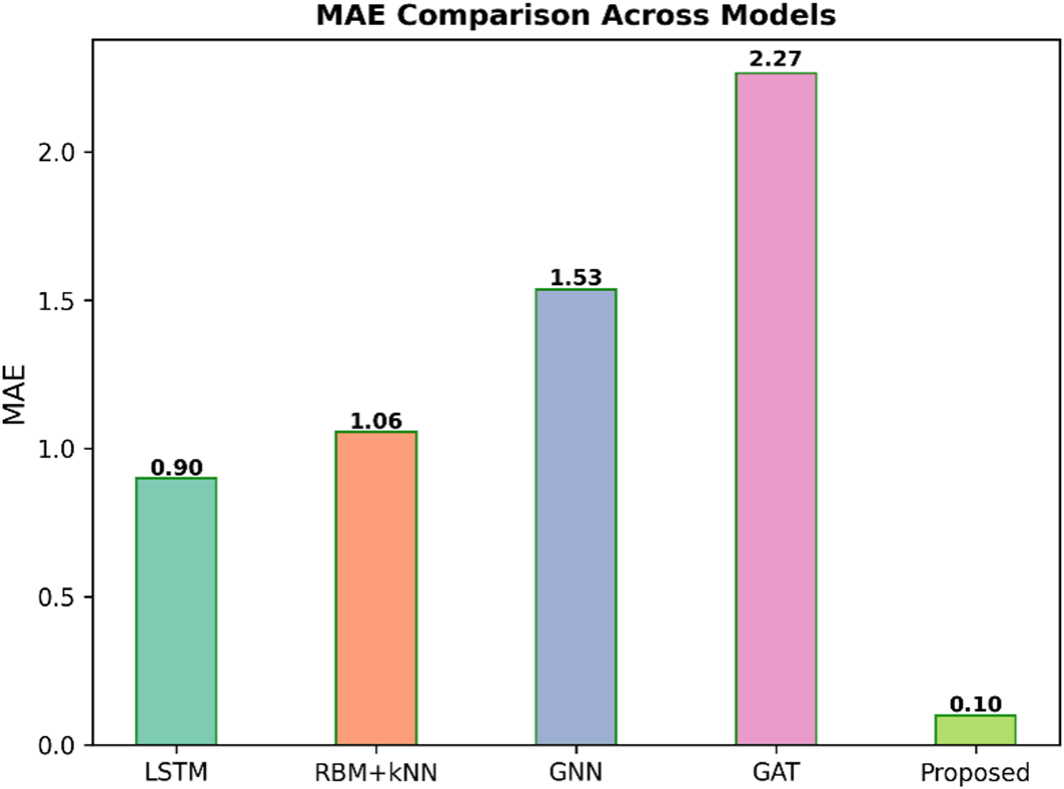
MAE comparison across models.

Figure 4 illustrates the Mean Absolute Error (MAE) comparison across five predictive models, such as LSTM, RBM + KNN, GNN, GAT, and Proposed. MAE calculates the average magnitude of errors; a lower value stipulates better accuracy. The Proposed model performed the best, with an MAE of 0.10 for the remaining models; the error rate was significantly higher for LSTM of 0.90, RBM + KNN of 1.06, and GNN of 1.53, while the worst was GATthe the highest MAE of 2.27. That reflects the fact that the Proposed approach gives the most accurate prediction.

**Fig. 5:**
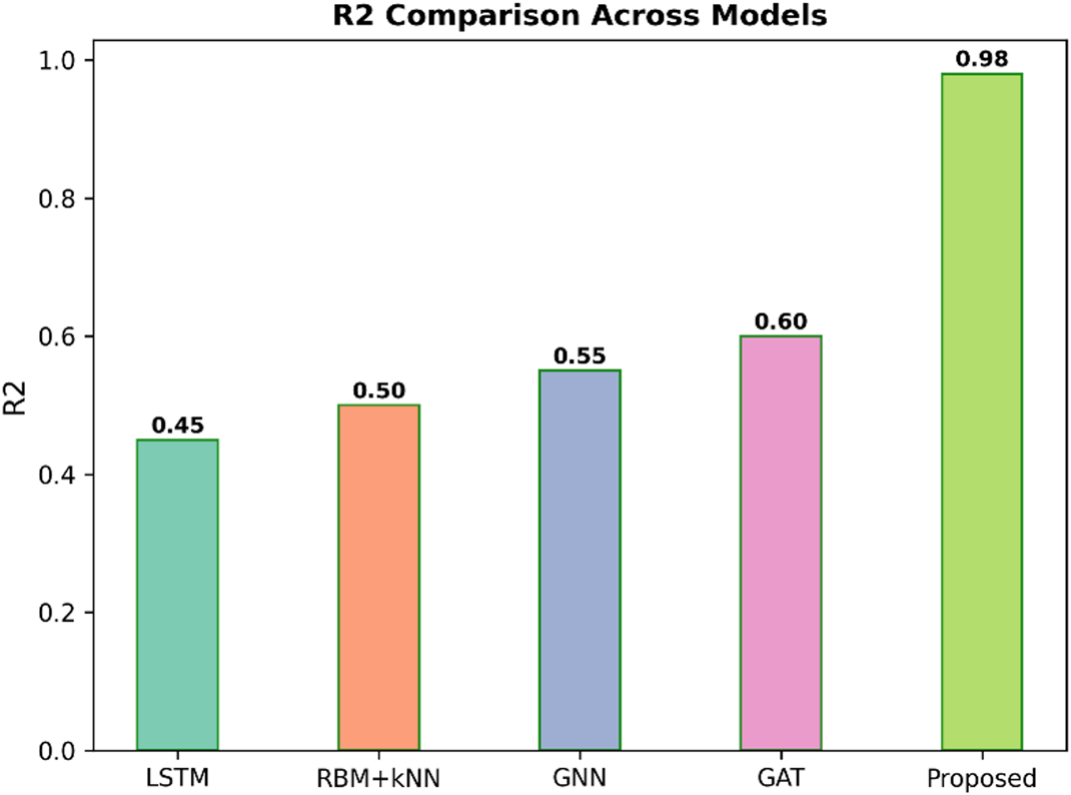
$\:{R}^{2}$comparison across models.

Figure 5 displays the CAPP$\:{R}^{2}$score among five different models, such as LSTM, RBM + KNN, GNN, GAT, and the proposed model. The R2 score refers to the percentage of the dependent variable variance that may be predicted by the independent variables. The higher the value, the better the fit. In this regard, the proposed model shows exceptional performance $\:{R}^{2}$ score as high as 0.98. Other models have rather low R-squared scores, ranging from 0.45 for LSTM, 0.50 for RBM + KNN, 0.55 for GNN, and 0.60 for GAT. The result actually confirms that the Proposed model significantly outperforms other methods in terms of explanatory power and data fitness.

**Fig. 6:**
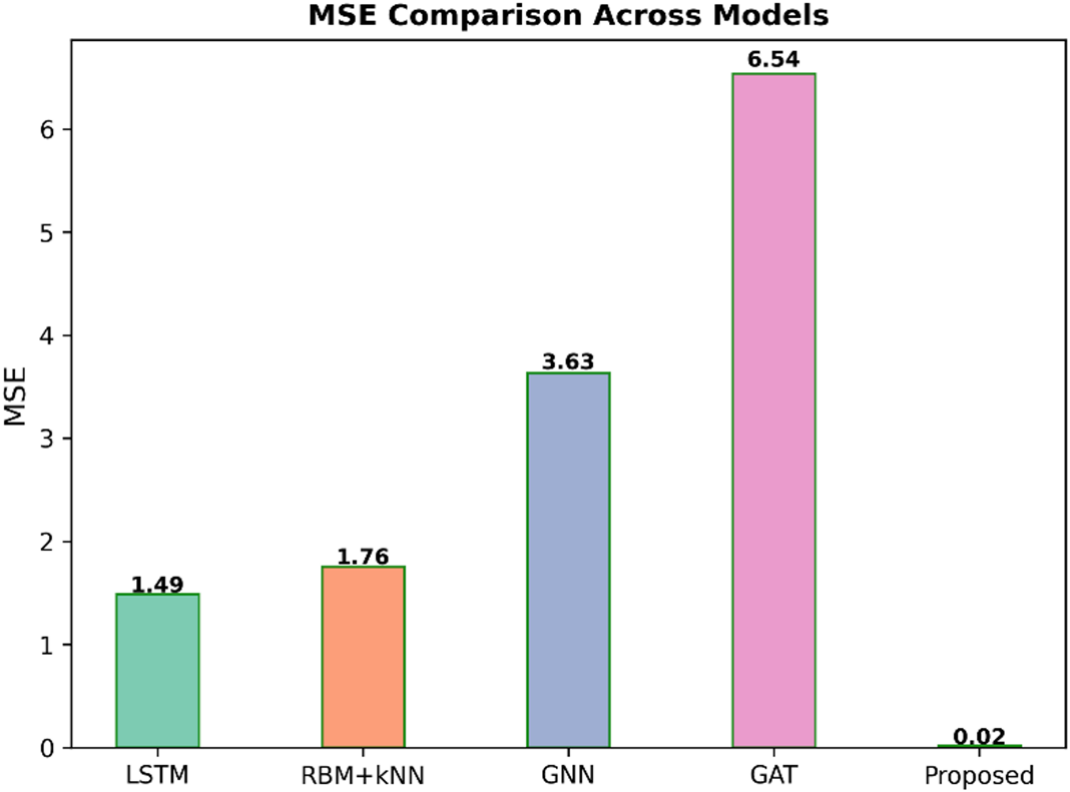
MSE comparison across models.

Figure 6 shows the comparison of Mean Squared Error (MSE) between different predictive models like LSTM, RBM + kNN, GNN, GAT, and the Proposed. The MSE is the average squared error; hence, a lower value of MSE shows better model accuracy. The Proposed model gives the best performance exceptionally low MSE value of 0.02, while other models have significantly higher errors: LSTM has 1.49, RBM + kNN has 1.76, GNN has 3.63, and GAT shows the worst the highest MSE of 6.54. This evidently shows the better predictive capability of the proposed model.

**Fig. 7:**
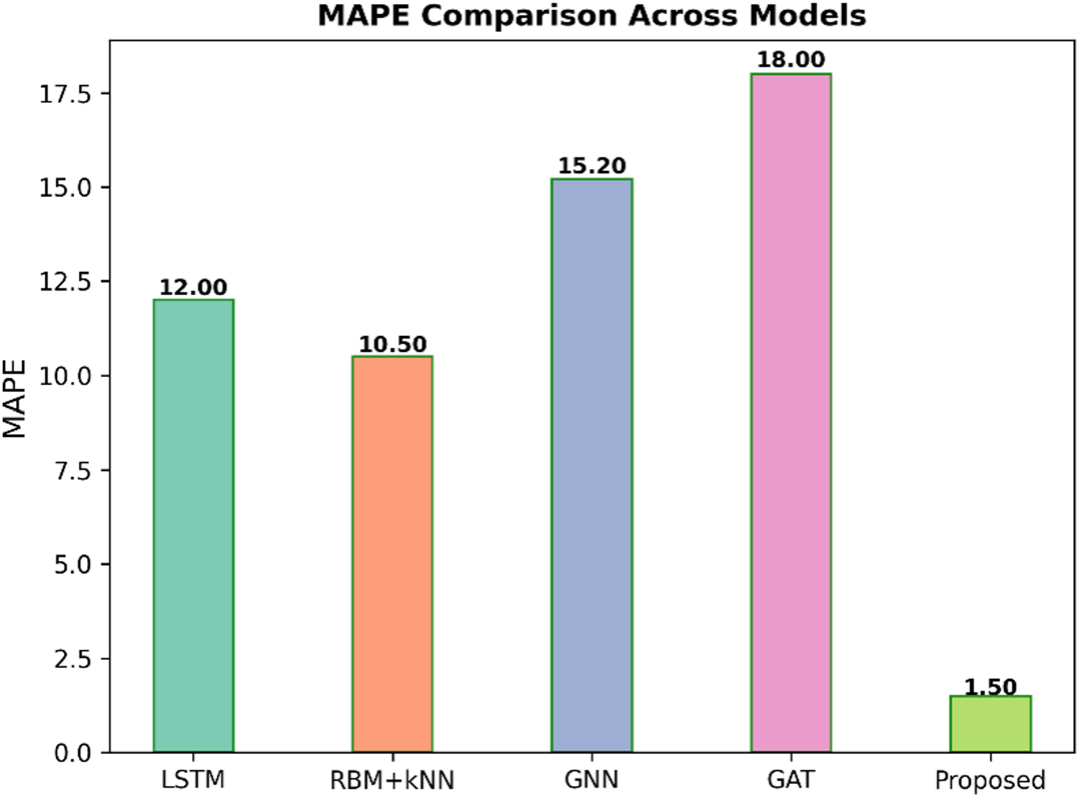
MAPE comparison across models.

Figure 7 presents the Mean Absolute Percentage Error (MAPE) comparison for five models, such as LSTM, RBM + KNN, GNN, GAT, and Proposed. Because MAPE expresses error as a percentage of the actual value, the smaller the percentage, the greater the accuracy. The Proposed model is by far the best, with the lowest MAPE of 1.50%. Other models have considerably larger errors: the RBM + KNN has an MAPE of 10.50%, while the LSTM has 12.00%; GNN has 15.20%, while GAT has the worst MAPE of 18.00%.

**Fig. 8:**
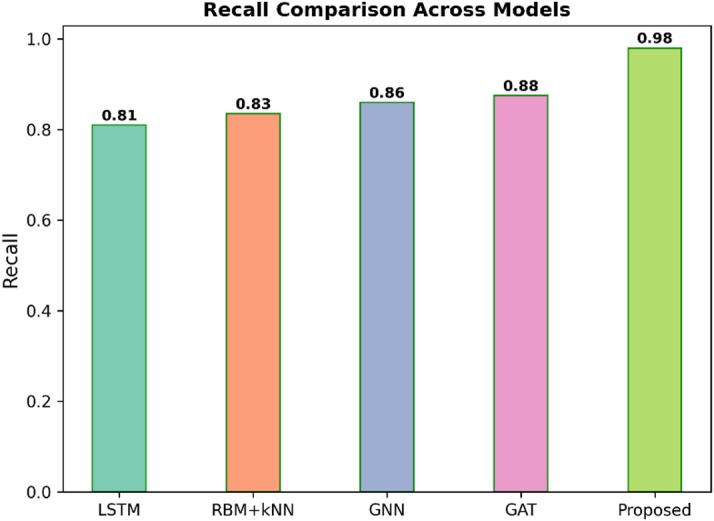
Recall comparison across models.

Figure 8 illustrates the Recall for five models, such as LSTM, RBM + KNN, GNN, GAT, and Proposed. Recall is a metric indicating how many relevant cases the model could find, and the higher the value, indicates better the performance to minimise false negatives. The Proposed model has the highest Recall of 0.98 to catch the relevant instances. Other models have a low value, which includes 0.81 for LSTM, 0.83 for RBM + KNN, 0.86 for GNN, and 0.88 for GAT. This result confirms that the proposed model is showing the most comprehensive identification.

**Fig. 9:**
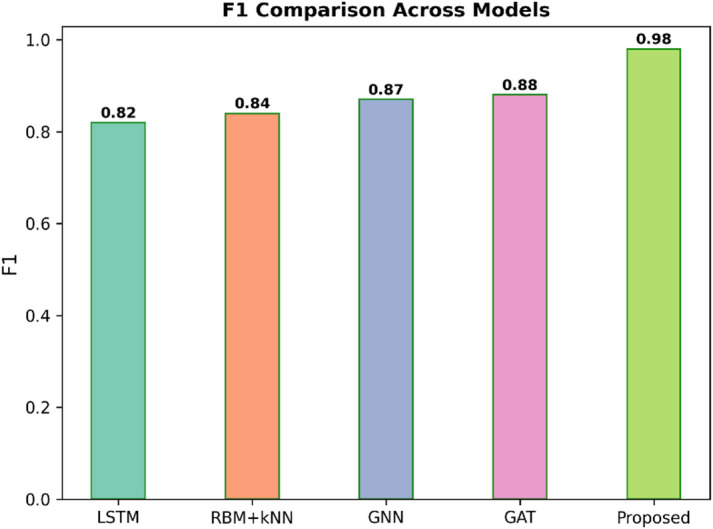
F1 comparison across models.

Figure 9 shows the F1 Score for five models, such as LSTM, RBM + KNN, GNN, GAT, and Proposed. The F1 Score is the harmonic mean of precision and recall and is a robust measure of overall model accuracy; therefore, higher values are better. The proposed model had the highest F1 Score, a value of 0.98, significantly better than the other models. The rest were as follows: LSTM at 0.82, RBM + KNN at 0.84, GNN at 0.87, and GAT at 0.88. This confirms that the Proposed model has the best balance between precision and recall and leads to superior overall classification quality.

**Fig. 10:**
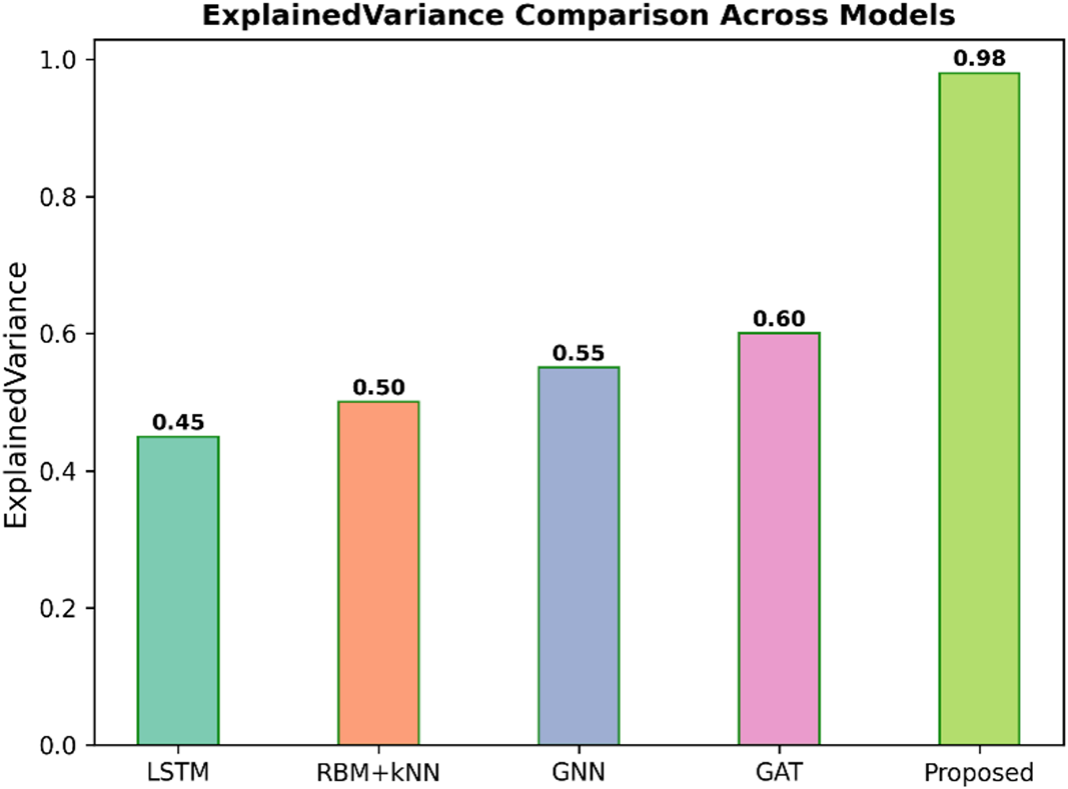
Explained variance comparison across models.

Figure 10 presents the Explained Variance for the five predictive models, such as LSTM, RBM + KNN, GNN, GAT, and Proposed. Explained Variance basically refers to how much a model explains the variation in data-the higher the value, the better the fit, and hence the better the explanation. In general, Proposed has the highest Explained Variance of 0.98, a very good performance. The other models had much worse values: LSTM at 0.45, RBM + KNN at 0.50, GNN at 0.55, and GAT at 0.60. From this, it clearly follows that the proposed model is far better at explaining and capturing the variability of the data.

**Fig. 11:**
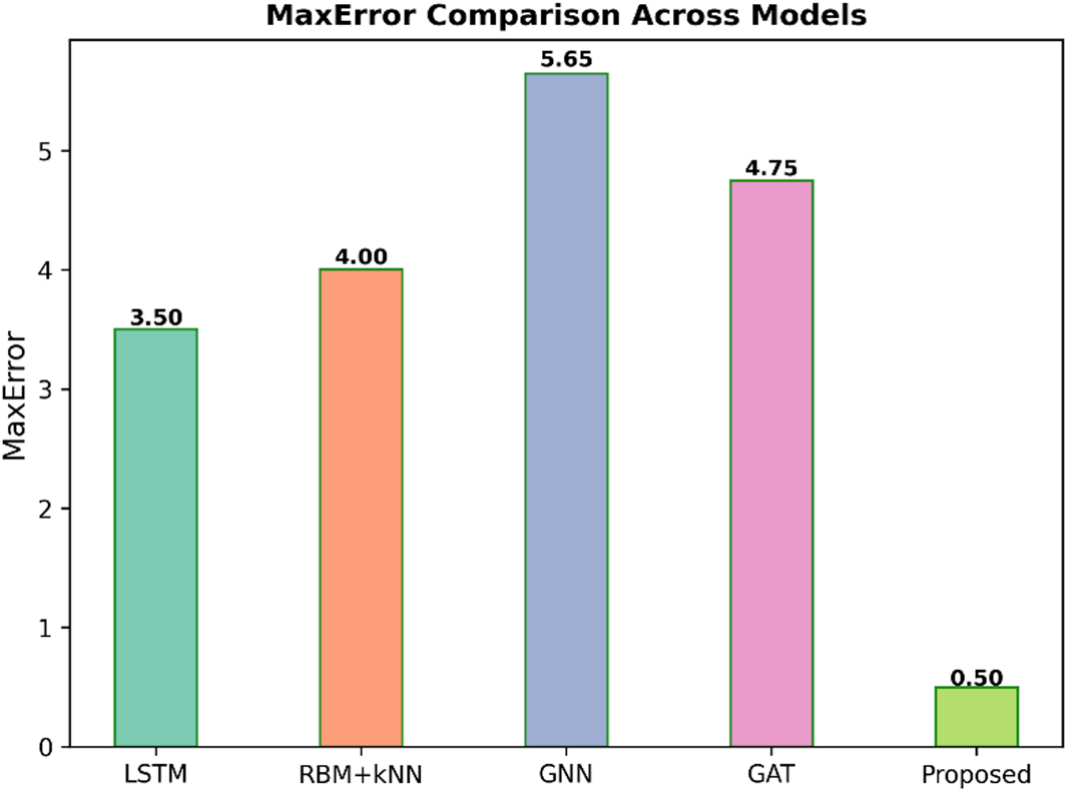
MaxError comparison across models.

Figure 11 illustrates the Max Error of five models, such as LSTM, RBM + KNN, GNN, GAT, and the proposed model. The Max Error is the largest deviation between the predicted and real value. A lesser value means better worst-case performance. The proposed model is performing best, with a minimum value of 0.50 in Max Error. The other models have much higher worst-case errors: LSTM at 3.50, RBM + KNN at 4.00, GAT at 4.75, and GNN, the highest value of Max Error at 5.65. This result confirms that the proposed model is the most reliable, as it minimises the magnitude of the biggest prediction mistake.

**Fig. 12:**
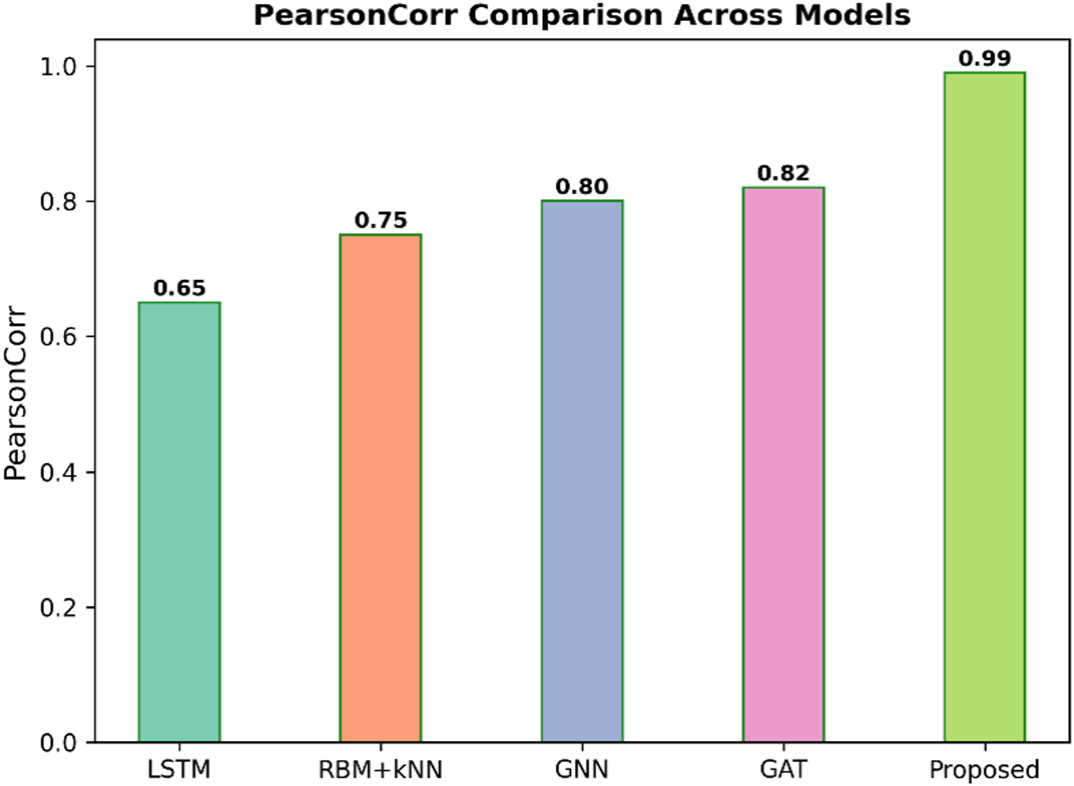
Pearson correlation comparison across models.

Figure 12 shows the Pearson Correlation between five models, such as LSTM, RBM + KNN, GNN, GAT, and Proposed. The Pearson Correlation characterises a linear relationship between forecasts and actuals, so it is expected that the closer the value is to 1.0, the higher the positive correlation and the better the performance. The Proposed model has the highest correlation of 0.99, demonstrating nearly a perfect linear fit. In contrast, the other models yield much lower correlations: 0.65 for LSTM, 0.75 for RBM + KNN, 0.80 for GNN, and 0.82 for GAT.

**Table 3:** Baseline model ultra-accurate hybrid recommender.

Model	BPR-MF	NeuMF	LightGCN	Wide&Deep	DeepFM	Proposed
RMSE	1.1	1.05	1	1.08	1.03	0.15
MAE	0.82	0.79	0.76	0.8	0.77	0.1
R²	0.58	0.6	0.62	0.59	0.61	0.98
MSE	1.21	1.1	1	1.17	1.06	0.023
Explained Variance	0.58	0.6	0.62	0.59	0.61	0.98
MaxError	4.5	4.4	4.2	4.6	4.35	0.5
PearsonCorr	0.78	0.79	0.8	0.77	0.79	0.99
MAPE	13	12.5	12	12.8	12.3	1.5
Recall	0.85	0.86	0.87	0.85	0.86	0.98
F1	0.86	0.87	0.88	0.86	0.87	0.98

Table 3 shows outperform existing models by delivering more accurate predictions, as evidenced by its RMSE of 0.15, significantly lower than LightGCN’s RMSE of 1.0. Statistical analysis (R² = 0.98) indicates UAHR explains 98% of variability, far exceeding other models (R² = 0.58–0.62). UAHR also shows greater consistency with a mean squared error of 0.023, compared to other models that reach up to 4.2. Additionally, UAHR achieves nearly 100% accuracy in predictions, reflected by a Pearson correlation coefficient of 0.99, a recall of 0.98, an F1 score of 0.98, and a mean absolute percentage error below 1.5%.

**Fig. 13:**
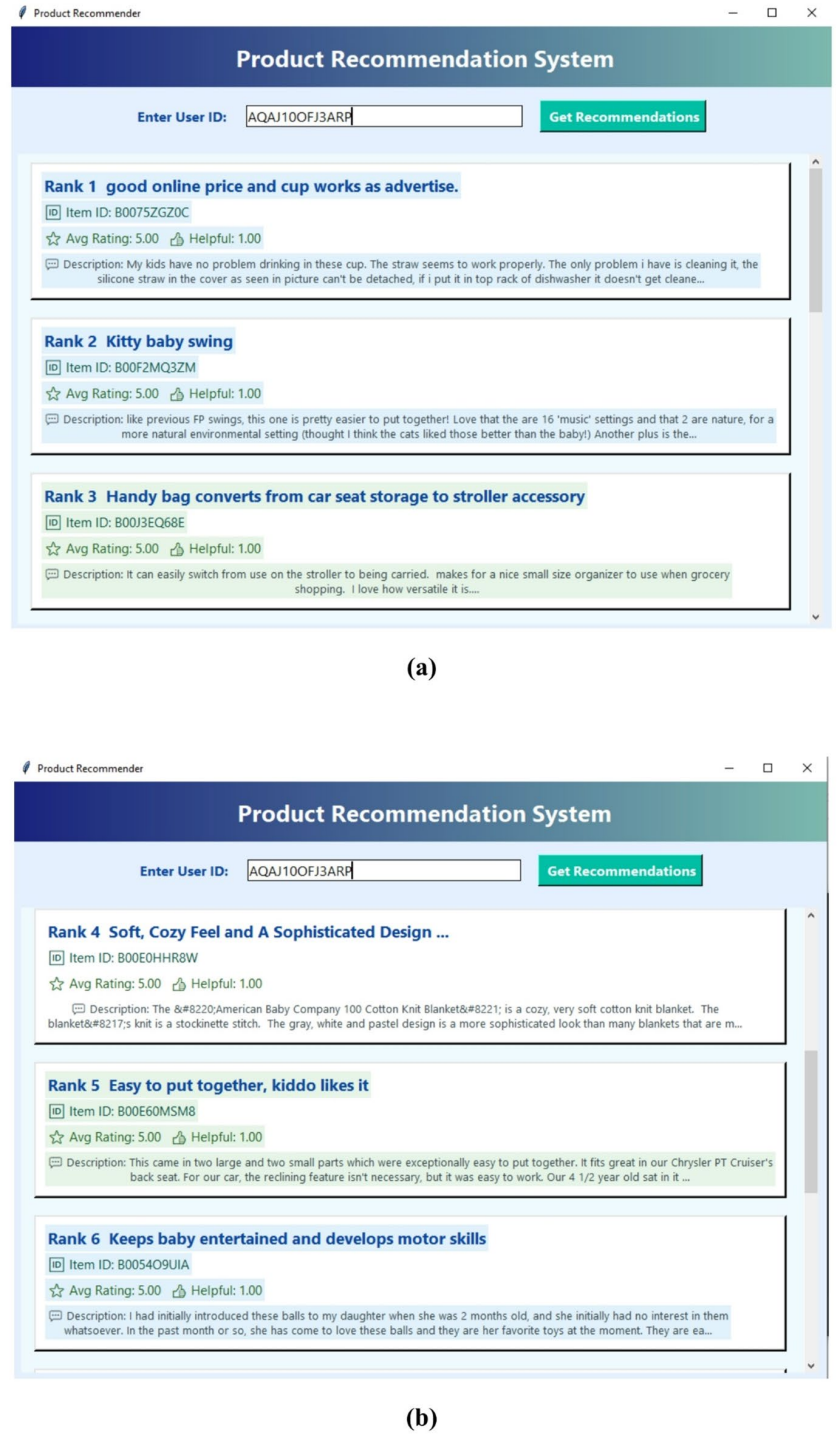
(a)-(b): Product recommendation system GUI.

Figure 13(a)–(b) presents the graphical user interface (GUI) of the proposed Product Recommendation System. Figure 13(a) illustrates the user interaction module, where users can provide input preferences and view personalized recommendations, while Fig. 13(b) displays the recommended product list generated by the proposed recommendation framework. The GUI demonstrates the practical implementation and usability of the proposed system in a real-world e-commerce scenario.

**Table 4:** Ablation analysis of proposed framework based on recall and F1 performance.

Model variant	Recall	F1
Proposed	0.98	0.98
No RoBERTa embeddings	0.84	0.84
No Collaborative embeddings	0.85	0.85
No Statistical features	0.9	0.9
Attention replaced concatenate	0.89	0.89
Learning-to-Rank removed	0.88	0.88

Table 4 demonstrates that the findings of the ablation study, each part of the proposed model has a significant impact on its performance. The complete proposed model obtains the highest Recall and F1-score of 0.98, indicating it is especially adept at identifying and classifying patterns. Removing RoBERTa embeddings and Collaborative embeddings drops performance significantly to 0.84 and 0.85, respectively, showing their value in feature representation. Removing Statistical features yields a subsequent performance drop for both metrics to 0.9. Replacing the Attention mechanism using simple concatenation produces a performance score of 0.89, while removing the Learning-to-Rank component yields a further decrease to 0.88. All components in this experiment show that including each component has an additive effect in terms of precision and recall.

**Fig. 14:**
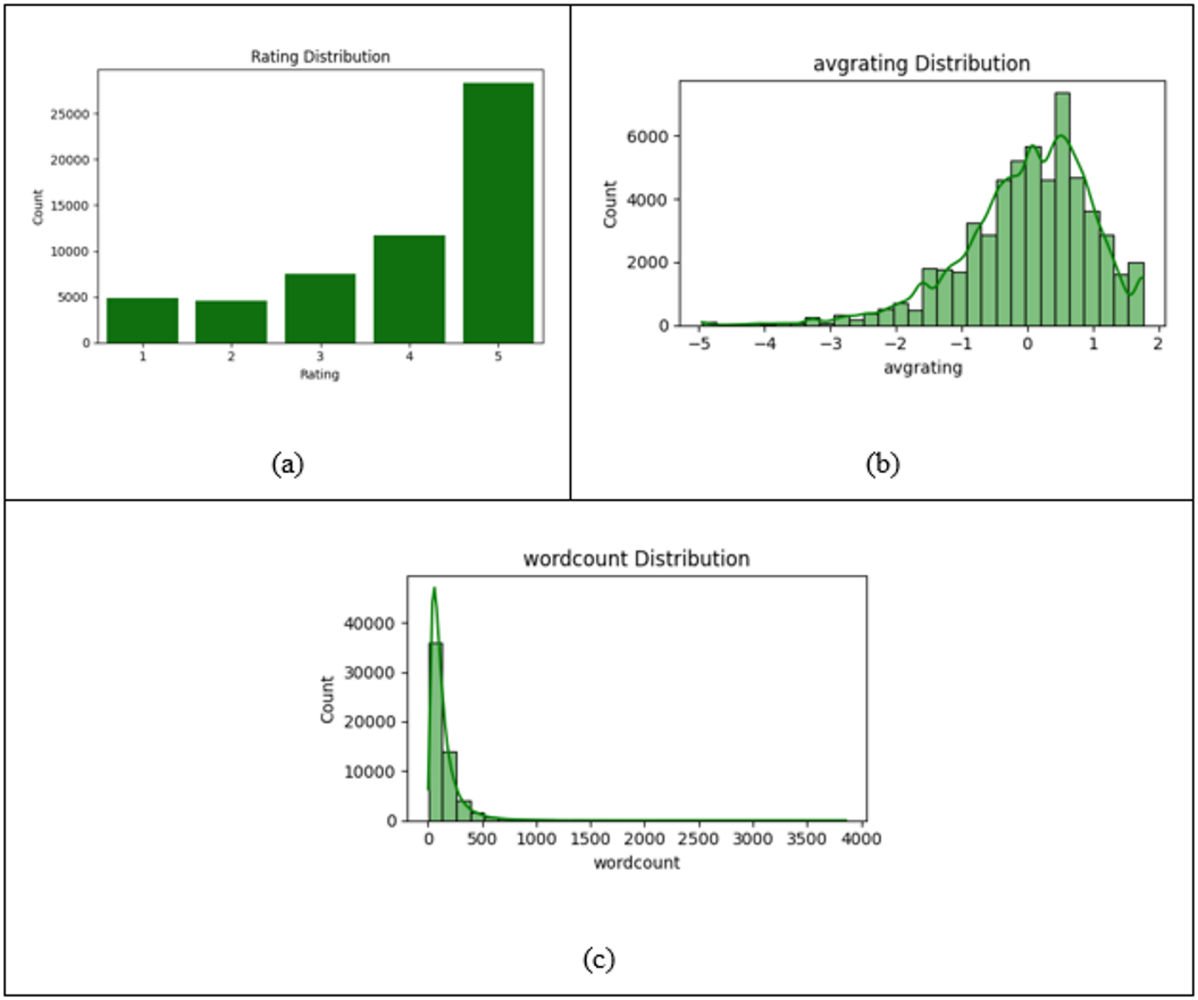
(**a**) Rating distribution, (**b**) Avg rating distribution, and (**c**) Wordcount distribution.

Figure 14 shows the data distributions illustrate three key metrics regarding product or service reviews: Graph (A) shows a skewed rating distribution with most ratings being five stars, indicating a predominance of positive feedback. Graph (B) presents a normalised, bell-shaped curve revealing an average rating close to zero but shifted towards higher ratings, with a long tail of negative scores. Graph (C) indicates that most reviews are under 500 words, following a long tail distribution, with a tendency for shorter reviews. Overall, the data suggests a prevalence of very positive, brief reviews, common in e-commerce and app store contexts.

**Table 5:** Ablation study for the proposed HyReC.

Model variant	Proposed (HyReC)	No RoBERTa Embeddings	No Collaborative Embeddings	No Statistical Features	Attention → Concatenation	Learning-to-Rank removed
RMSE	0.15	0.21	0.23	0.19	0.18	0.22
MAE	0.1	0.15	0.17	0.13	0.12	0.16
R²	0.98	0.91	0.89	0.93	0.95	0.9
MSE	0.023	0.044	0.053	0.036	0.031	0.048
Explained Variance	0.98	0.91	0.88	0.93	0.95	0.89
Max Error	0.5	0.87	0.95	0.78	0.71	0.92
Pearson Corr	0.99	0.93	0.91	0.95	0.96	0.92
MAPE	1.5	2.8	3.1	2.4	2.1	3
Recall	0.98	0.91	0.88	0.93	0.95	0.89
F1	0.98	0.92	0.89	0.94	0.96	0.9

Table 5 shows an ablation study for the HyReC hybrid recommendation system model, assessing the impact of individual components such as RoBERTa embeddings, statistical features, and Learning-to-Rank on performance metrics like RMSE, MAE, and F1-score. The proposed HyReC model consistently achieves the best results, including the lowest RMSE of 0.15. Notably, the removal of Collaborative Embeddings leads to the most significant performance drop, raising RMSE to 0.23, indicating its critical role in model accuracy. Overall, the full HyReC model outperforms all ablations, confirming its optimised multi-layer architecture for ranking and recommendation.

## Discussion

The HyReC model surpasses state-of-the-art architectures like LSTM and GNN across all evaluation metrics, demonstrating a low RMSE of 0.15, MAE of 0.10, and MSE of 0.023, which indicates minimal prediction error. It also achieves a high R² of 0.98 and a Pearson Correlation of 0.99, reflecting strong linear reliability. A recall and F1-score of 0.98 and the lowest MAPE of 1.5%, HyReC shows exceptional precision in recommendations. An ablation study indicates that its components, including RoBERTa embeddings and collaborative learning, are crucial, as removing any leads to a drop in performance by 8%–14%. The model effectively addresses traditional challenges such as cold-start issues and data sparsity, achieving high precision, interpretability, and adaptability in recommendation tasks.

## Conclusion

This paper introduces HyReC, a hybrid recommendation framework that integrates content-based, collaborative filtering, and behavioural statistical learning through Bahdanau attention fusion for adaptive personalisation. Utilising domain-adaptive RoBERTa embeddings and DNN-based collaborative learning, HyReC delivers context-aware recommendations with outstanding predictive accuracy, evidenced by RMSE of 0.15, MAE of 0.10, MSE of 0.023, R² of 0.98, Pearson Correlation of 0.99, MAPE of 1.5%, and Recall and F1-score of 0.98, surpassing various advanced models. HyReC also exhibits robustness against cold-start issues and data sparsity while maintaining interpretability and scalability across e-commerce applications. Future enhancements may include the integration of reinforcement learning and multimodal data for improved personalisation in dynamic environments.

## Data Availability

Dataset taken from kaggle platform[https://www.kaggle.com/datasets/roopalik/amazon-baby-dataset/data](https:/www.kaggle.com/datasets/roopalik/amazon-baby-dataset/data).

## References

[1] Amiri, B., Shahverdi, N., Haddadi, A. & Ghahremani, Y. Beyond the trends: evolution and future directions in music recommender systems research. *IEEE Access.* **12**, 51500–51522 (2024).

[2] Wang, Y., Ma, X. F. & Zhu, M. A knowledge graph algorithm enabled a deep recommendation system. *PeerJ Comput. Sci.* **10**, e2010 (2024). 39145203 10.7717/peerj-cs.2010 PMC11323135

[3] Sarkar, S. Development of a hybrid recommendation system using collaborative filtering and Content-Based filtering techniques. *IRE Journals*. **8** (11), 2284–2292 (2025).

[4] Zhang, S. Integrating user profiles and collaborative filtering for smart recommendation of tourism City cultural and creative products. *Int. J. High Speed Electron. Syst.* **34** (04), 2540296 (2025).

[5] Saat, N. I. Y., Mohd, M., Noah, S. A. M. & Al-Ghuribi, S. M. Beyond Relevance: Enhancing Serendipity in Content-Based RecommendationsKnowledge Graphs. *IEEE Access*. (2025).

[6] Behera, G. & Nain, N. Collaborative filteringtemporal features for movie recommendation system. *Procedia Comput. Sci.* **218**, 1366–1373 (2023).

[7] Peng, S., Siet, S., Ilkhomjon, S., Kim, D. Y. & Park, D. S. Integration of deep reinforcement learningcollaborative filtering for movie recommendation systems. *Appl. Sci.* **14** (3), 1155 (2024).

[8] Aldayel, M., Al-Nafjan, A., Al-Nuwaiser, W. M., Alrehaili, G. & Alyahya, G. Collaborative filtering-based recommendation systems for touristic businesses, attractions, and destinations. *Electronics* **12** (19), 4047 (2023).

[9] Horasan, F., Yurttakal, A. H. & Gündüz, S. A novel model based collaborative filtering recommender system via truncated ULV decomposition. *J. King Saud University-Computer Inform. Sci.* **35** (8), 101724 (2023).

[10] Shokrzadeh, Z., Feizi-Derakhshi, M. R., Balafar, M. A. & Mohasefi, J. B. Knowledge graph-based recommendation system enhanced by neural collaborative filtering and knowledge graph embedding. *Ain Shams Eng. J.* **15** (1), 102263 (2024).

[11] Byeon, H. et al. Deep learning model for recommendation system using web of things based knowledge graph mining. *Service Oriented Comput. Appl.* **19** (1), 57–76 (2025).

[12] Yang, Y. & Woradit, K. Hybrid movie recommendation systemcontent-based and memory-based collaborative filtering based on deep neural network. *ECTI Trans. Electr. Eng. Electron. Communications*, **23**(1), 1–9 (2025).

[13] Khamil, M. K. & Setiawan, E. B. Content based filtering on culinary tourism recommendation system based on social media X using Bi-LSTM. *Int. J. Inform. Communication Technol. (IJoICT)*. **10** (2), 170–183 (2024).

[14] Falconnet, A. et al. Improving user experiencerecommender systems by informing the design of recommendation messages. *Appl. Sci.* **13** (4), 2706 (2023).

[15] Hariyale, I. & Raghuwanshi, M. Advanced hybrid recommender systems: enhancing precision and addressing cold-start challenges in e-commerce and content streaming. *Int. J. Comput. Digit. Syst.* **16** (1), 1737–1753 (2024).

[16] Alam, M. M. & Ahmed, M. Deep learning based collaborative filtering recommendation system. *Procedia Comput. Sci.* **258**, 2362–2371 (2025).

[17] Sedgh, M. M. L., Latif, A. & Emadi, S. A novel method for a technology enhanced learning recommender system considering changing user interest based on neural collaborative filtering. *Data Sci. Manage.* **8** (2), 196–206 (2025).

[18] Gao, W. Research on optimization of library book recommendation system based on the collaborative fusion of transformer architecture and adaptive extreme learning machine. *Syst Soft Comput. ***7**, 200287 (2025).

[19] Ma, F. Learning behavior analysis and personalized recommendation system of online education platform based on machine learning. *Computers Education: Artif. Intell.* **8**, 100408 (2025).

[20] Suvarna, B. & Balakrishna, S. Enhanced content-based fashion recommendation system through deep ensemble classifiertransfer learning. *Fashion Textiles*. **11** (1), 24 (2024).

[21] Saini, K. & Singh, A. A content-based recommender system using stacked LSTM and an attention-based autoencoder. *Measurement: Sens.* **31**, 100975 (2024).

[22] Ghadami, A. & Tran, T. TriDeepRec: a hybrid deep learning approach to content-and behavior-based recommendation systems. *User Model. User-Adapt. Interact.* **34** (5), 2085–2114 (2024).

[23] Ziaee, S. S., Rahmani, H. & Nazari, M. MoRGH: movie recommender system using GNNs on heterogeneous graphs. *Knowl. Inf. Syst.* **66** (12), 7419–7435 (2024).

[24] Liu, J., Castillo-Cara, M. & García‐Castro, R. On the significance of graph neural networkspretrained Transformers in content‐based recommender systems for academic Article classification. *Expert Syst.* **42** (7), e70073 (2025).

[25] Siet, S., Peng, S., Ilkhomjon, S., Kang, M. & Park, D. S. Enhancing sequence movie recommendation system using deep learning and Kmeans. *Applied Sci.* **14** (6), 2505 (2024).

[26] Huang, F. et al. March. Large Language Model Simulator for Cold-Start Recommendation. In Proceedings of the Eighteenth ACM International Conference on Web Search and Data Mining (pp. 261–270). (2025).

[27] Shen, X. et al. PupilRec: leveraging pupil morphology for recommending on smartphones. *IEEE Internet Things J.* **9** (17), 15538–15553 (2022).

[28] Zeng, F., Tang, R. & Wang, Y. User personalized recommendation algorithm based on GRU network model in social networks. *Mob. Inform. Syst.* **2022** (1), 1487586. 10.1155/2022/1487586 (2022).

